# Advances in the intraoperative delineation of malignant glioma margin

**DOI:** 10.3389/fonc.2023.1114450

**Published:** 2023-01-26

**Authors:** Shan Jiang, Huihui Chai, Qisheng Tang

**Affiliations:** Department of Neurosurgery, Huashan Hospital, Fudan University, Shanghai, China

**Keywords:** glioma, intraoperative, MRI, FGS, Raman histology, mass spectrometry

## Abstract

Surgery plays a critical role in the treatment of malignant glioma. However, due to the infiltrative growth and brain shift, it is difficult for neurosurgeons to distinguish malignant glioma margins with the naked eye and with preoperative examinations. Therefore, several technologies were developed to determine precise tumor margins intraoperatively. Here, we introduced four intraoperative technologies to delineate malignant glioma margin, namely, magnetic resonance imaging, fluorescence-guided surgery, Raman histology, and mass spectrometry. By tracing their detecting principles and developments, we reviewed their advantages and disadvantages respectively and imagined future trends.

## Introduction

At present, the standard treatment for malignant glioma is surgical resection combined with chemotherapy and radiotherapy, which is far from reaching patients’ expectations and offers slow progress (1–3). As the first step, surgery plays a critical role in multimodal treatments and its efficacy is highly dependent on the surgeon’s skill. Moreover, due to the infiltrative growth, it is difficult to depict the tumor margin and excise the tumor completely (4). Consequently, it contributes to a high local relapse rate, and most recurrences occur near the surgery margins. Therefore, delineating more sophisticated brain tumor margins and improving surgeons’ ability to navigate removing the tumor completely are important for the improvement of brain tumor treatments (5).

Therefore, to visualize the tumor margin and assist neurosurgeons in resecting tumors completely, establishing a precise and real-time guiding system has already become an active demand in neurosurgery. Besides the infiltrative growth nature, the requirement of protecting brain functional boundaries emphasizes more importance to improving the resolution of margin demarcation than other solid tumors in the peripheral system. Various techniques in detecting tumor molecular or metabolic markers by physical or chemical methods allow for depicting millimeter-level resolution boundaries (6, 7). Moreover, due to the impact of brain shifting, it is necessary for various techniques to be performed and to amend intraoperative detection (8–10).

**Figure 1:**
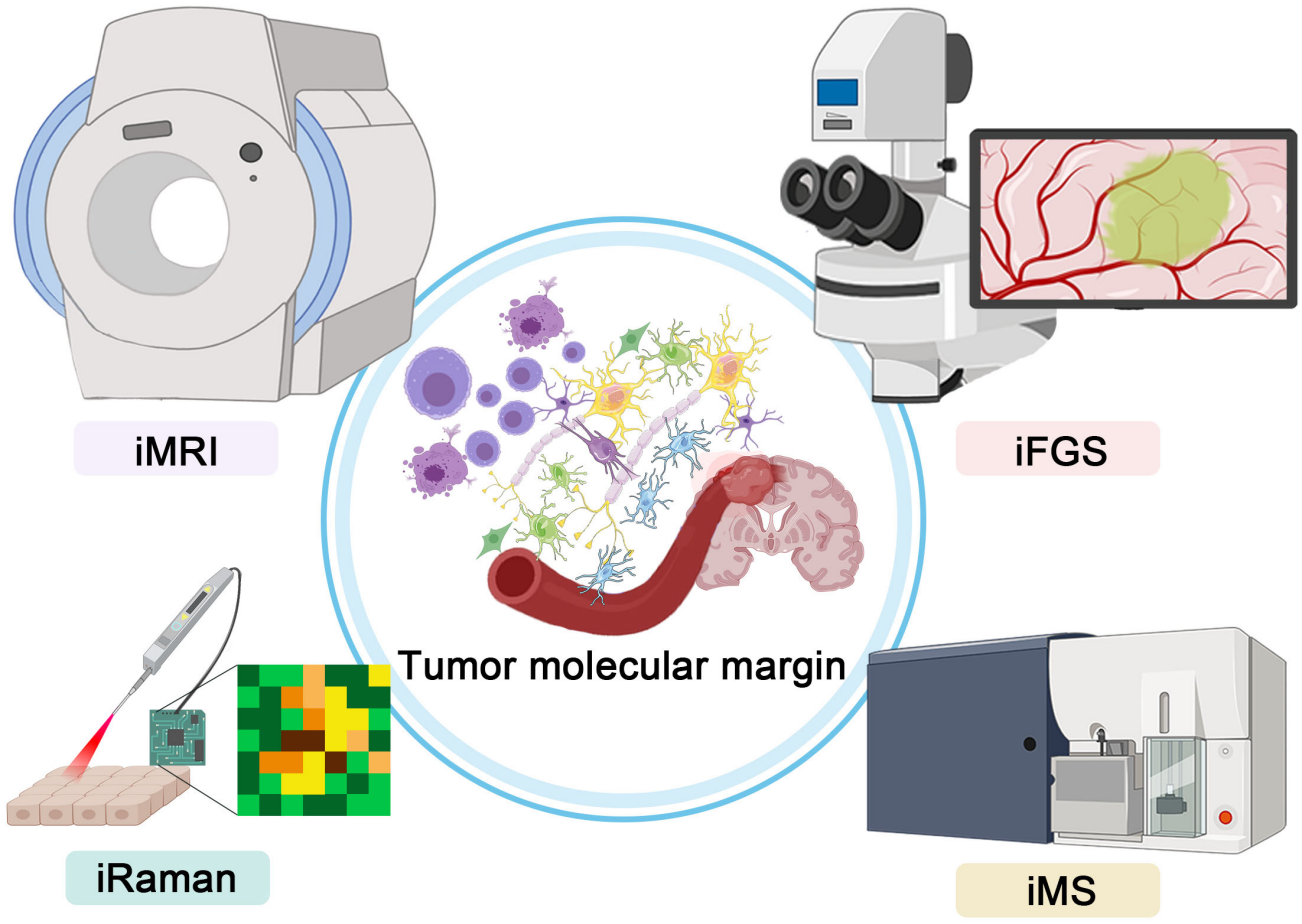
Graphical abstract showing the four intraoperative technologies for precise depictions of malignant glioma margin.

Here, we list the developments of several novel intraoperative technologies depicting precise malignant brain tumor margins, that is, magnetic resonance imaging (MRI), fluorescence-guided surgery (FGS), Raman histology, and mass spectrometry (MS) (**Figure 1**). Analyzing the advantages and disadvantages of different technologies, we provide a comprehensive review to trace the updated developments of new intraoperative systems.

## Intraoperative MRI

MRI is the essential preoperative examination for brain cancer patients and a salient basis for intraoperative navigation. However, due to the presence of brain shift, which is inevitably caused by the loss of cerebrospinal fluid, force-induced deformation of brain tissue, and so on, the utilization of preoperative image data to guide neuronavigation will inevitably lead to deviations (11). These deviations may lead to either residual tumor after resection or over-resection of normal brain tissue. Though various software for correction algorithms based on physical or mathematical models are under research and development, clinical examinations of this software are required (12, 13).

Currently, intraoperative imaging remains the established solution (14). Contributing to its high contrast in soft tissues, precise spatial resolution, and functional brain imaging ability, intraoperative MRI (iMRI) becomes the first option among various intraoperative imaging systems (15, 16). The first public application of iMRI in the neurosurgical community was reported by General Electric and Brigham and Women’s Hospital (BWH) of Harvard University in 1996 (17, 18). The past 26 years have witnessed a great development in iMRI systems. Previous generations of iMRI were based on moving the operating table into the MRI examination room. Nowadays, fundamental innovations in the engineering and physics of the magnet and coils allow the movement of MRI scanners. The common characteristic of the newest iMRI systems is the performance of intraoperative imaging without the movement of patients, that is, to be able to perform surgery while the scan is being completed and with the patient in the same position (19). The field strength of iMRI is also increasing, which improves the imaging quality. Moreover, high field strength provides the advantages of less signal acquisition time, high sensitivity of functional imaging, and quantitative analysis of tissue metabolites based on spectrum signal (20, 21). Senft et al. performed a prospective, randomized, parallel-group trial to confirm the efficacy of iMRI application. They found that 96% (23/24) of the patients in the iMRI group and 68% (17/25) of the patients in the control group had complete tumor resection. No patient for whom the use of iMRI led to continued resection of the residual tumor had neurological deterioration. It indicated that imaging helped surgeons provide the optimum extent of resection (22). Huashan Hospital retrospectively analyzed 373 patients with 3T iMRI-guided surgery. The ratio of gross total resection for cerebral gliomas (*n* = 161) was increased from 55.90% to 87.58% (23). Also, Kuhnt et al. proved the correlation between the extent of tumor resection and glioblastoma multiforme patient survival with high-field iMRI, demonstrating that navigation guidance and iMRI significantly contribute to optimal EOR with low postoperative morbidity, where the extent of resection ≥98% and patient age <65 years are associated with significant survival advantages (22). However, high-field-strength iMRI has its share of problems. Owing to the interference of waves caused by the dielectric effect, the imaging signal is uneven and the center signal is higher than the peripheral one. Also, compared with 1.5 T iMRI, motion artifacts, chemical shift artifacts, and susceptibility artifacts were more obvious on 3.0 T iMRI (24).

Besides the structure imaging ability of iMRI, its functional imaging and molecular imaging show more and more advantages in the operation of malignant glioma. The protection of brain function during operation is a very salient issue in neurosurgery. Different from the peripheral system with more clear structural markers, individual brain functional regions have great differences in details. Therefore, electrophysiological monitoring and intraoperative arousal techniques play critical roles in the resection of tumors located in brain functional areas. Usually, preoperative MRI examination uses blood oxygen level-dependent imaging (BOLD) and diffusion tensor imaging (DTI) technologies to achieve fMRI imaging of brain functional areas, to achieve relatively intuitive and gradual delineation of brain functional area boundaries, and to help design surgical approaches and resection schemes. Lehéricy et al. and Wu et al. both reported that BOLD localization of the motor cortex was in good agreement with the results of controlled studies on direct electrical stimulation during surgery (25–27). Studies by Rutten et al. show that BOLD and electrical stimulation technology have good consistency in locating the language cortex (28). However, the delineation of malignant glioma functional margin based on preoperative MRI has its limitations. Because of the disturbance in the operation, the accuracy of the preoperative functional partition decreases with the operation. The tiny deviations can have serious consequences when it comes to the functional brain regions. Fortunately, iMRI can achieve both fMRI and DTI. Besides the localization of the motor cortex using the task-based intraoperative fMRI technique during awake procedures, the resting state localizing the motor cortex of patients who are under general anesthesia could be detected using intraoperative fMRI (29, 30). Also, D’Andrea et al. reported that they localized white matter tracts participating in language with intraoperative DTI instead of direct electrical cortical stimulation (DCS) and that 78% of patients achieve gross total excision without any postoperative complications (31). However, the inherent flaws in the functional detection ability of MRI may result in limited statistical power, arbitrary data analysis, false-positive results, and lack of independent replications (32). Therefore, though iMRI could improve the precision of fMRI, the fact that it is time-consuming and unstable makes it unlikely to replace DCS, while intraoperative DTI has the potential to increase the safety and excision extension of non-awake surgery.

Not only functional imaging, but also molecular imaging might be a promising development direction. In 2021, the fifth edition of the WHO Central System Tumor Grading Criteria highlighted the importance of tumor molecular markers as an important basis for classification. As for preoperative MRI, it has already shown its potential in detecting the qualitative, quantitative, and localization of specific molecular chemicals. Magnetic resonance spectroscopy (MRS) is a technology that uses the chemical shift of atomic nucleus caused by the external magnetic field to realize a non-invasive *in vivo* study of physiological or pathological metabolic changes. The hydrogen proton (^1^H-MRS) is the most commonly used one. Compared with conventional MRI, ^1^H-MRS can determine the nature and value-added activity of lesions from the aspect of metabolism (33). In the process of occurrence and development of many diseases, the metabolic changes are earlier than the pathological changes (34). Therefore, MRS can distinguish and classify gliomas at the biochemical level (35–37). Roder et al. reported the feasibility of intraoperative MRS and its potential usage in an extended tumor resection (38). However, due to the low spatial resolution, the long examination time, and interference of fat and skull, there is slow progress in the application of intraoperative MRS during surgery. Currently, the mainstream application is to register preoperative MRS imaging into intraoperative MRI, so as to achieve more targeted biopsy or excision guidance (39, 40). More convincing research evidence is needed to support the necessity of carrying out intraoperative MRS.

The application of iMRI also has other limitations. The utilization of iMRI significantly increases operative time compared with traditional operation. It requires additional time for setup, registration, draping, and redraping of patients, as well as the transport of the patient into and out of the scanner (41, 42). It is necessary to take into consideration the additional time needed for patient selection and the scheduling of the operating room. Lastly, the huge cost of iMRI equipment purchase ranges from $3 million to $7 million, not including the inconvenient cost of renovating the operative suite (43). Meanwhile, considerable costs are required for the maintenance of iMRI instruments and to employ specialized staff to assist in surgery. Though there was a study demonstrating the potential economic advantages of less hospital stay and lower total hospital costs from using iMRI (44), there is no doubt that the high cost of purchasing an iMRI system has become a major hindrance to universal implementation (43, 45).

Overall, the iMRI system has already been widely applied and exhibits a fantastic prospect. Its high spatial resolution and the depiction of brain function and metabolism margin improve the effect of surgical removal of brain tumors. The future development of iMRI relies on the amelioration of devices and computational performance, including higher magnetic field intensity, higher gradient performance, and multi-channel signal acquisition. The digitally integrated neurosurgical operation center based on iMRI could contribute to interactively integrating a variety of minimally invasive new technologies, to achieve less surgical trauma and more complete surgical resection. Compared with mechanical design, the effect of algorithms and high-performance computers would play a more important role. It is possible to enhance the spatiotemporal resolution of iMRI without the need for magnetic field strength improvement (46, 47). Besides the imaging resolution of brain structures, it is mainly used for determining functional and metabolic boundaries to guide more sophisticated surgical resection.

## Intraoperative fluorescence

Another approach to maximize the extent of resection and avoid neurological damage is to use fluorescence dyestuff to highlight tumor regions and improve visual contrast. In recent decades, several fluorescence contrast agents, such as 5-aminolevulinic acid (5-ALA), indocyanine green (ICG), and fluorescein sodium (FLS), have been available for intraoperative use in clinical settings (48, 49).

5-ALA is the precursor of heme synthesis, which can generate protoporphyrin IV (PpIV) during metabolism (50). PpIV has strong photosensitive activity. In malignant tumor cells, the activity of enzymes involved in PpIV production is stronger than that in normal cells, while the activity of enzymes catalyzing the conversion of PpIV to hemochrome is weaker than that in normal cells. Therefore, a large amount of PpIV is accumulated in tumor cells (51, 52). Using this characteristic, the PpIV-rich tumor tissue can emit red fluorescence (635–705 nm) after being irradiated by an appropriate spectrum (407 nm) during the operation. Thus, tumors can be distinguished from normal tissues under the microscope. Therefore, 5-ALA has the advantages of convenient administration (oral administration before surgery), favorable visualization, and repeated administration (53, 54). It was reported that the sensitivity and specificity of 5-ALA in dense HGG tissue were above 90% (55). However, different studies show varying sensitivities (from 21% to 95%) and specificities (from 53% to 100%) (56–60). Meanwhile, 5-ALA is prone to show false-positive results in low-grade glioma, edema, and inflammatory tissues, and the fluorescence signal of deep tumors may also be covered by normal tissues (61–63).

FLS is a fluorescent compound used for obtaining diagnostic biopsies initially. It could be excited by light in the 460- to 500-nm range and send out the yellow-green part of the spectrum between 540 and 690 nm (64). Its application in neurosurgery was pioneered by Moore et al. in 1947 (65). FLS could be intravenously injected and visualized in the tumor tissue through the blood–brain barrier (66). Since the development of microscopes, the dose of FLS frequently used has been 1–2 mg/kg, guaranteeing safety and tolerability (67). Because of its low cost and simple operation, FLS is easy to promote in clinical practice (68). However, it is not directly bound to glioma cells but only accumulates in the tumor tissue; thus, the specificity is lower than that of 5-ALA (69, 70).

## Intraoperative Raman histology

Raman histology is a label-free imaging method that uses intrinsic biochemical markers to distinguish tumor tissues (71, 72). In 1928, the Indian physicist Raman found that the inelastic scattering phenomenon occurs when the frequency of an incident photon is shifted after being scattered by a molecule. Therefore, it is possible to identify specific agents according to their unique Raman scattering spectrum (73). Since tumor cells have the ability to change metabolism to promote their rapid proliferation, Raman histology testing the chemical changes (e.g., protein, lipids, nucleic acids, and pH) could assess the tumor margin to guide the resection, based on the chemical composition of both inorganic and biological specimens. Hollon et al. reported that the diagnostic accuracy using stimulated Raman histology in 48 glioma patients was 95.8%. They concluded that the utilization of stimulated Raman histology improved the intraoperative detection of glioma recurrence in near-real time (74).

Considering its characteristics of real-time and rapid detection, intraoperative Raman histology has already shown its potential to achieve real-time molecular pathological level of tumor boundary detection (75). Surface-enhanced Raman scattering (SERS) is a spectroscopic technique based on the plasmon-assisted scattering of molecules absorbed on the noble metal surface, which has high photostability, sensitivity, and potential for the simultaneous detection of up to 10 compounds (76, 77). Jin et al. reported an intelligent SERS Navigation System to guide brain tumor resection. They detected tumor tissues’ metabolic acidosis (pH 6.2–6.9) caused by glucose metabolism shifting from oxidative phosphorylation to aerobic glycolysis. The efficiency had been examined in both animal models and patients. This system accelerates the clinical translation of acidic margin-guided surgery and avoids exogenous imaging probes. Also, concerning its detection of extracellular acidification, which is a common marker in solid tumors and does not rely on specific genetic phenotypes, SERS depiction of solid tumor margin testing metabolic acidosis might have a broader and universal application prospect (78).

However, intraoperative Raman histology has its drawbacks. Firstly, through the spatial resolution at the millimeter level, a small area of each detection range (mm^2^) increased the number of repeated operations and the workload of neurosurgeons (79). Meanwhile, *ex vivo* imaging requires tissue removal, which also limits its further clinical application (80). The future development of Raman histology depends on the feasibility of rapid wide-ranging examination and instrument combination with integration and miniaturization.

## Intraoperative mass spectrometry

Mass spectrometry is a method of detecting moving ions by separating them according to their mass-to-charge ratios using electric and magnetic fields. The composition of ions can be determined by measuring the exact mass of ions. It is widely used in the laboratory to qualitatively and quantitatively analyze the composition, molecular phenotype, and content of the samples, which has the advantages of high detection speed and high sensitivity (81). The commonly used indicators of glioma detected by mass spectrometry include N-acetylaspartic acid (NAA), 2-hydroxyglutaric acid (2-HG), choline, creatine (Cr), myoinositol (mI), lactic acid (Lac), and lipid (Lip). According to quantitative analysis of certain contents, neurosurgeons could not only distinguish tumor tissue from normal brain tissue but also identify molecular subtypes of brain tumors (82, 83). For example, the content of NAA reflects the number of neurons and axons, and the concentration of NAA often decreases significantly in brain tumor tissues. 2-HG is the metabolite of *isocitrate dehydrogenase* (*IDH*) gene mutant glioma; the concentration in *IDH-mutant* tumor samples could be more than 100 times the size of a normal brain tissue (84, 85). The detection of such chemical indicators by intraoperative mass spectrometry provided the ability to characterize the molecular and metabolic boundaries of tumors. Considering its operating principles, the technique applies to a wide range of chemicals and promises high-throughput analysis.

Moreover, in 2004, desorption electrospray ionization mass spectrometry (DESI-MS) made it possible to direct intraoperative sampling analysis without the need for sample preprocessing (86). In 2015, Alan et al. reported that the DESI-MS detections of 158 glioma samples, 223 gray matter samples, and 66 white matter samples could effectively distinguish glioma from white matter and gray matter, and the overall sensitivity and specificity reached 97.4% and 98.5%, respectively (87). Moreover, with the development of techniques, the detection time is reduced to 3 min according to Pirro et al.’s report in 2017 (88).

In a nutshell, intraoperative mass spectrometry has shown the potential to redefine the maximum resection of glioma. Despite its rapid development, the clinical application of iMS remains limited in the operating room, which is essential to complete validation of large-sample-size data. The limitation of spatial resolution and detection accuracy required the development of the ionization technique. The miniaturization of intraoperative simple testing instruments and the establishment of rapid intraoperative testing procedures are the key to accelerate clinical popularization. Also, future development requires more stable biomarkers, a mass spectrometry library, and integrated equipment.

## Discussion

We summarized different intraoperative techniques for delineating the precise boundaries of brain tumors, including MRI, fluorescence-guided surgery, Raman histology, and mass spectrometry. Considering their inability to depict precise boundaries, especially molecular and metabolic margins, we did not include intraoperative computerized tomography and ultrasound in this review. The principle and development of each technique are briefly introduced, and their advantages and disadvantages are analyzed. Regardless of the differences in imaging methods, the ultimate goal of accurate delineation of tumor molecular boundaries is to improve the gross total resection and the surgical benefits for brain tumor patients on the premise of protecting normal brain tissues. At the same time, the delineation of tumor molecular boundaries is helpful to further improve the judgment of molecular classification of brain tumors and guide the subsequent diagnosis and treatment. Though we illustrated them respectively, different techniques were not mutually exclusive; for example, intraoperative mass spectrometry could also be used in conjunction with intraoperative MRI (89). The necessity and priority of the use of different techniques need to be explored and verified. Also, how to interact and fuse multiple methods to form multimodal delineation of tumor boundaries is an important direction for future research and development.

At present, the application of these new technologies is hindered by their high cost. ElGamal et al. have analyzed the effectiveness and cost-effectiveness of intraoperative fluorescence, intraoperative ultrasound, and intraoperative MRI (90). All approaches have been shown to significantly improve the gross total resection and progression-free survival of high-grade gliomas, while the high cost of these new techniques has significantly hindered the progress and prevalence of these techniques. Therefore, cost reduction and the improvement of integrated and miniaturized intraoperative detection equipment are urgent problems that need to be solved in future engineering and manufacturing development. To promote the popularization of these new technology, it requires to be compatible with common operating rooms. One possible solution to strike balance between accuracy and economic efficiency is to establish a large-sample multimodal database and use artificial intelligence algorithms to improve the original imaging resolution (91–93).

Based on the criteria of intraoperative real-time imaging and delineation of tumor boundaries at the molecular level, we selected intraoperative MRI, fluorescence-guided surgery, Raman histology, and mass spectrometry for review. Due to the rapid development of technology and the differences in different regions, inevitably, the tracking of new technologies is not timely. Based on physical and chemical principles, advances in mechanical engineering and computer technology make it possible to apply these theories to delineate tumor molecular boundaries in operating rooms. We firmly believe that these multidisciplinary advances and integrated applications drive the continuous progress of medical treatment. The continuous cultivation of these methods also continuously updates our understanding of cancer and innovates our diagnosis and treatment methods, to effectively protect the life and health of patients.

## Author contributions

SJ wrote the first draft of the manuscript. HC revised the manuscript and was in charge of language editing. QT reviewed and edited the final manuscript. All authors contributed to the article and approved the submitted version.
